# Development of Real-Time Immuno-PCR Based on Phage Displayed an Anti-Idiotypic Nanobody for Quantitative Determination of Citrinin in *Monascus*

**DOI:** 10.3390/toxins11100572

**Published:** 2019-09-30

**Authors:** Wenping Huang, Zhui Tu, Zhenqiang Ning, Qinghua He, Yanping Li

**Affiliations:** State Key Laboratory of Food Science and Technology, Jiangxi-OAI Joint Research Institute, Jiangxi Province Key Laboratory of Modern Analytical Sciences, Nanchang University, Nanchang 330047, China; huangwenping1110@163.com (W.H.); tuzhui@ncu.edu.cn (Z.T.); zhenqiang-ning@seu.edu.cn (Z.N.); heqinghua@ncu.edu.cn (Q.H.)

**Keywords:** citrinin, mimotope, anti-idiotypic nanobody, phage display, real-time immuno-PCR

## Abstract

Citrinin (CIT) is a mycotoxin that has been detected in agricultural products, feedstuff, and *Monascus* products. At present, research has been performed to develop methods for CIT detection, mainly through TLC, HPLC, biosensor, and immunoassay. The immunoassay method is popular with researchers because of its speed, economy, simplicity, and ease of control. However, mycotoxins are inevitably introduced during the determination. Immunoassays require the use of toxins coupled to carrier proteins or enzymes to make competitive antigens. In this study, anti-idiotypic nanobody X27 as CIT mimetic antigen was used as non-toxic surrogate reagents in immunoassay. Therefore, the X27-based real-time immuno-PCR (rtIPCR) method had been established after optimal experiments of annealing temperature and amplification efficiency of real-time PCR, concentration of coating antibody, phage X27, and methyl alcohol. The IC_50_ value of the established method in the present study is 9.86 ± 2.52 ng/mL, which is nearly equivalent to the traditional phage ELISA method. However, the linear range is of 0.1–1000 ng/mL, which has been broadened 10-fold compared to the phage ELISA method. Besides, the X27-based rtIPCR method has no cross-reactivity to the common mycotoxins, like aflatoxin B_1_ (AFB_1_), deoxynivalenol (DON), ochratoxin A (OTA), and zearalenone (ZEN). The method has also been applied to the determination of CIT in rice flour and flour samples, and the recovery was found to be in the range of 90.0–104.6% and 75.8–110.0% respectively. There was no significant difference in the results between the rtIPCR and UPLC–MS. The anti-idiotypic nanobody as a non-toxic surrogate of CIT makes rtIPCR a promising method for actual CIT analysis in *Monascus* products.

## 1. Introduction

Citrinin (CIT) is a secondary metabolite produced by certain species of the genus *Penicillium, Aspergillus,* and *Monascus* [1,2]. In the early stage of the discovery of CIT, it was highly valued by researchers because of its antibacterial activity [3,4]. However, subsequent animal experiments have shown that CIT has nephrotoxicity [5], hepatotoxicity, teratogenicity, carcinogenesis, and other negative effects on animal health, and CIT is therefore listed as a mycotoxin. The contamination of CIT is widespread and can occur in cereal agricultural products [6] and animal feed, such as wheat [7], barley [8], corn [9], rice [10,11], and cereal products [12]. In addition, its main producing strain, *Monascus,* produces a large amount of CIT during fermentation [13,14]. Therefore, food additives, medicines, and healthcare products prepared by various types of *Monascus* rice can cause pollution.

At present, many methods have been developed to detect CIT. TLC can be used to detect CIT [15], in which the adsorbent or the support agent is evenly spread on a glass plate, and then the sample of CIT to be separated is spotted on a thin layer and spread with a suitable organic solvent of CIT [16,17]. Gimeno et al. first used this method for the detection of CIT in apples, pears, juices, and jams with a minimum detection limit of 30–40 μg/kg [18]. The detection capabilities of HPLC vary depending on the type of sample, the method of pretreatment, and the type of detector. At present, mainstream detectors include ultraviolet detectors, fluorescence detectors, photodiode array detectors, and mass spectrometer detectors [19,20,21,22]. Biosensors are mainly composed of biological components and signal converters, which can amplify and convert biological signals of target molecules into electrical signals or other signals for analysis and detection purposes. This method can also be applied to quantitative determination of CIT [11,23,24,25]. These methods produce sensitive and reliable results but they are laborious, costly, and conducted in a sequential manner, making them unsuitable for routine analysis of a large scale of samples [26,27]. CIT is often analyzed by immunoassay because of its high specificity, accuracy, low cost, and convenience for high throughput screening [11,28]. Cheng et al. selected a CIT-specific single-chain antibody in a non-immune mouse single-chain antibody library and established an indirect competition ELISA with an IC_50_ value of 1.24 μg/mL and a linear range of 0.001–100 μg/mL [29]. Although most ELISA methods are sensitive, the use of organic solvents and standard can result not only in harm to the environment and human health, but also in poor reproducibility [30]. Therefore, it has been widely recognized that there is a need to develop reproducible and safe mycotoxin substitutes for use in immunoassays. Polypeptides or anti-unique nanobodies can be used as a substitute.

In our previous study, we built CIT mimotope from a naive alpaca heavy-chain single-domain antibody library [30]. Thirty random phage clones from the fourth round panning round were selected and identified by phage ELISA. Almost all competitive eluted heavy-chain single-domain antibody phages showed distinct levels of binding bio-activity to CIT monoclonal antibody. Only one clone, named X27, was considered as an anti-idiotypic nanobody, which exhibited inhibition binding activity to the primary antibody by free CIT standard [31]. In this study, we adopted phage X27 as the competitive antigen of CIT and combined the specificity of ELISA with the high sensitivity of real-time PCR to construct a new immunoassay method through optimization of PCR annealing temperature, concentration of coated monoclonal antibody, input phage concentration, and so on. Finally, a stable and reliable real-time immuno-PCR (rtIPCR) method was established for CIT detection in *Monascus* products.

## 2. Results

### 2.1. Verification of Correctness of Primers

**Figure 1:**
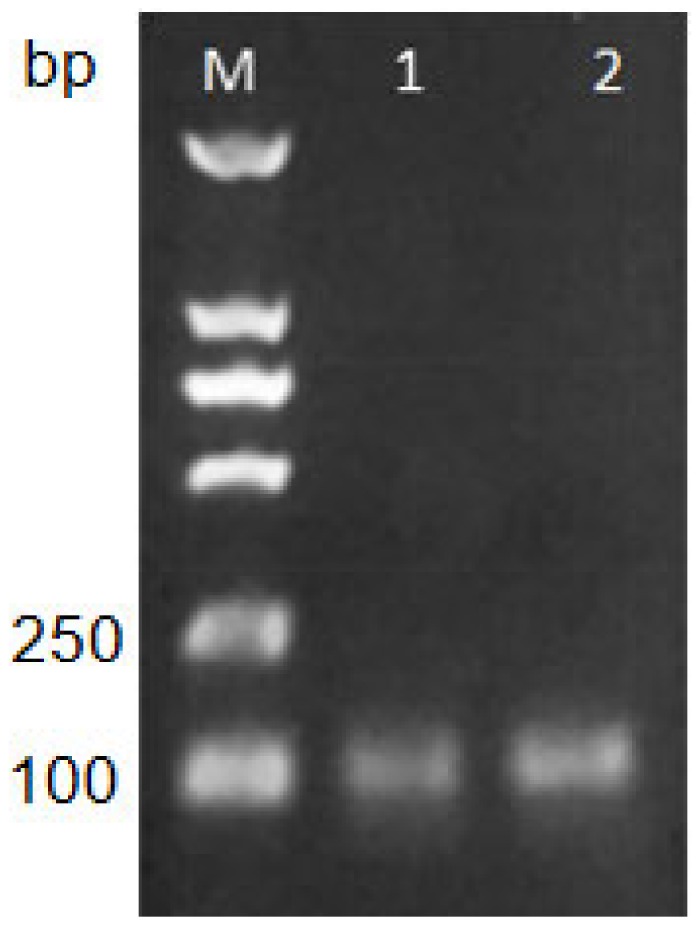
Analysis of PCR products with primers 219 and 220 by agarose gel electrophoresis. Lane M: DL2000 DNA Marker; Lane 1: Plasmid pHEN-X27; Lane 2: Phage X27.

In order to ensure the correctness of the primers for rtIPCR amplification, it was verified by PCR methods. The reaction product amplified by the rTaq enzyme was subjected to agarose gel electrophoresis and observed under the imaging system (Figure 1). The results show that a single target band was successfully amplified from plasmid pHEN-X27 and phage X27 with 219 and 220 as specific primers, and the fragment size was 100 bp, which was in line with expectations. Therefore, primers 219 and 220 can be used for further optimization experiments of rtIPCR.

### 2.2. Optimization of Real-Time PCR Reaction Conditions

**Figure 2:**
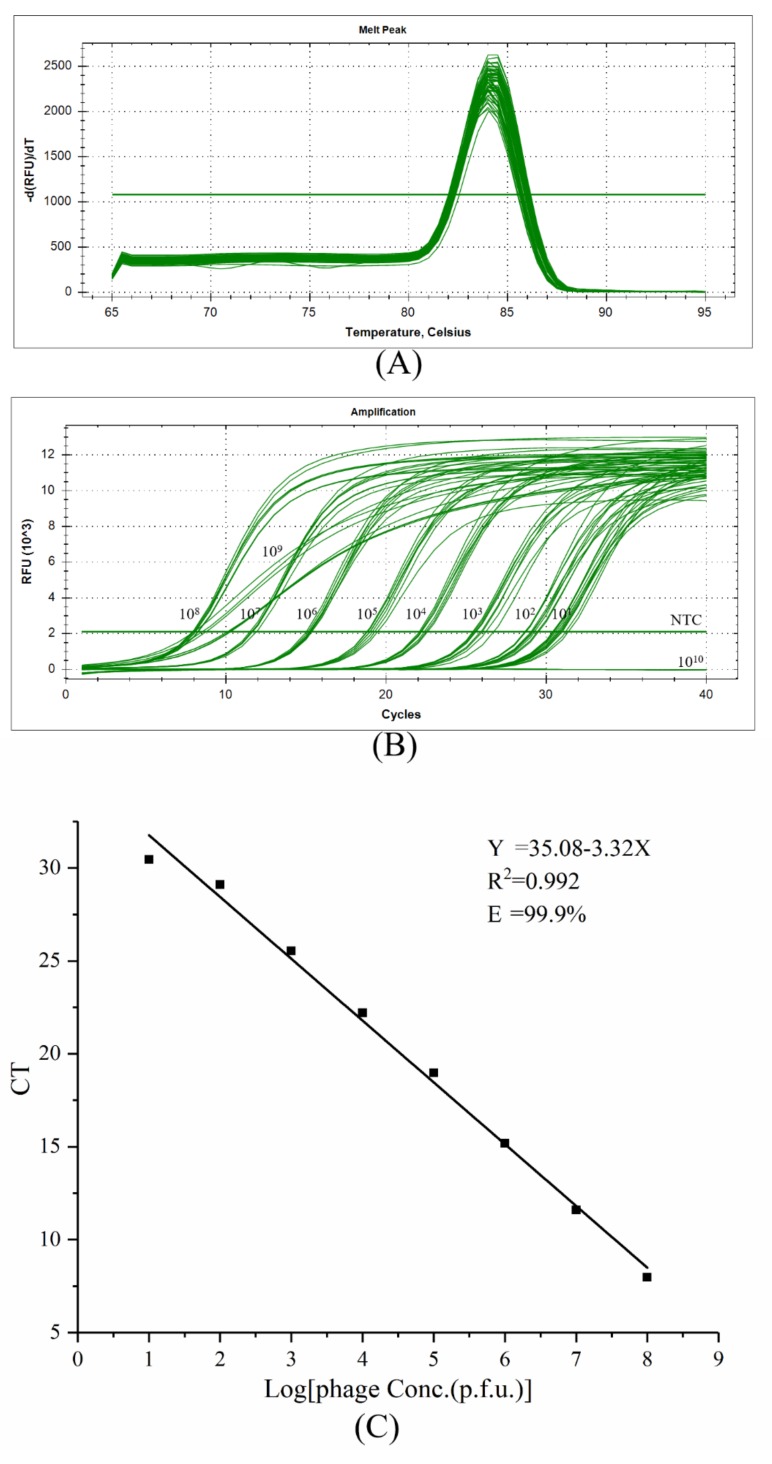
Optimizing real-time PCR reaction conditions. (**A**) Real-time PCR melt peak of phage X27. (**B**) Real-time PCR amplification curve of phage X27. (**C**) The standard curve of phage amplification with annealing temperature 62 °C.

To ensure the stability of the real-time PCR results, the phage concentration and annealing temperature were varied to optimize the amplification efficiency of the real-time PCR reaction. The titer of the amplified phage X27 was measured, and the result shows that the titer was 4 × 10^13^ pfu, and 10-fold serial dilutions of phage X27 were used as templates for PCR amplification. The results show that the specificity of the real-time PCR reaction was good and there was only a single peak in the melting curve (Figure 2A). From the results of the amplification curve (Figure 2B), the concentration of phage had a greater influence on the PCR reaction. When the number of phages was no less than 10^9^ pfu, PCR amplification was abnormal; if the number of phages was 10^10^ pfu, the PCR could not amplify the target band; if the number of phages was 10^9^ pfu, the PCR amplification efficiency was low; and if the number of phages was no more than 10^8^ pfu, PCR amplification was normal. Figure 2B shows different threshold cycles (C_T_s) at different phage numbers, and there is a linear relationship between phage concentration and C_T_ value. However, the annealing temperature had no significant effect on the real-time PCR reaction process, and there was almost no difference in C_T_ value between parallel samples (data not shown). The results show (Figure 2C) that the amplification efficiency was optimally 99.9% when the annealing temperature was 62 °C. The C_T_ values increased with the decrease of phage concentration. The standard equation was Y = 35.08 − 3.32X (Y: C_T_ value, X: Phage concentration), the correlation coefficient was *R*^2^ = 0.992, and the amplification efficiency was 99.9%. Therefore, 62 °C was chosen as the annealing temperature for rtIPCR.

### 2.3. Establishment of rtIPCR for CIT

#### 2.3.1. Optimization of the Concentration of Monoclonal Antibodies (McAbs)

**Figure 3:**
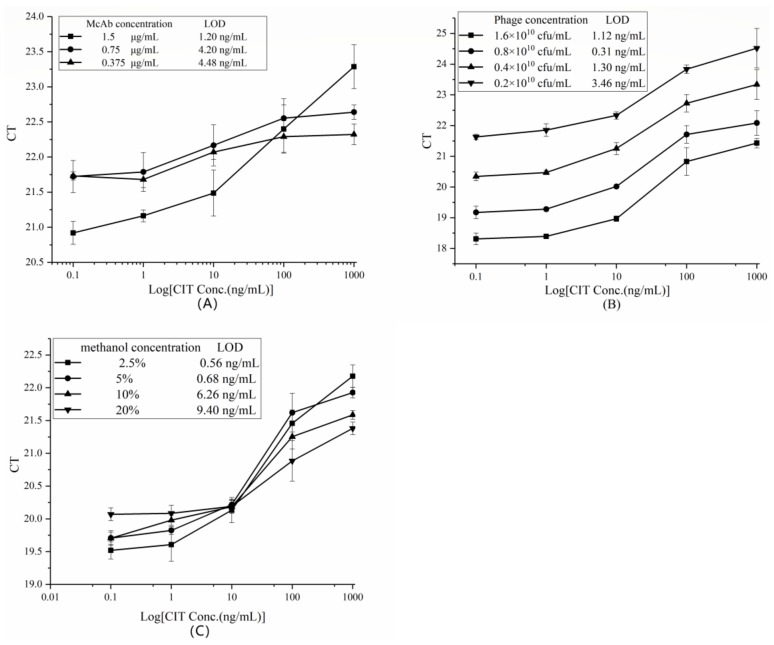
Optimization of rtIPCR for detection of CIT. (**A**) The optimization of concentration of anti-CIT McAb. (**B**) The optimization of concentration of phage X27. (**C**) The optimization of concentration of methanol.

Checkerboard procedure and followed fluorescent PCR were used to optimize coating antibody concentrations, phage amount, and methanol concentration. Different concentrations of anti-CIT McAb were adopted for rtIPCR at phage particle input of 6 × 10^8^ pfu and methanol concentration of 10%. The obtained C_T_ values and CIT standard concentrations were plotted as line graphs, and the LOD values were roughly calculated. Figure 3A shows that the LOD of the rtIPCR method was different at different concentrations of anti-CIT McAb. Among them, when the McAb concentration was 1.5 μg/mL, the corresponding LOD reached a minimum value of 1.20 ng/mL. Besides, at the same concentration of anti-CIT McAb, the difference of C_T_ values enlarged with the increase of CIT concentration, which is conducive to the establishment of a more stable and reliable analytical method. Therefore, the concentration of coated anti-CIT McAb of 1.5 μg/mL was selected for the next optimization experiment.

#### 2.3.2. Optimization of Phage Input

To optimize the amount of phage input, phage X27 was diluted to different multiples with sterile PBS for rtIPCR experiments. As shown in Figure 3B, the C_T_ values of the rtIPCR generally increased with the decrease of the phage input amount. At the same time, the LOD reached its minimum level of 0.31 ng/mL when the phage concentration was 0.8 × 10^10^ pfu. Therefore, this concentration was selected as the optimal phage input amount for subsequent optimization experiments.

#### 2.3.3. The Effect of Methanol Concentration

In this study, CIT was extracted from samples using 50% methanol in water. However, high methanol concentrations can interfere with protein activity and affect the binding capacity between antigen and antibody. To assess the influence of methanol, a series of dilutions of the CIT standard were dissolved in different methanol concentrations (2.5, 5, 10, and 20%) in PBS and subjected to rtPCR. When the methanol concentration was 20%, the linear range of the method was reduced to 10–1000 ng/mL and the LOD was 9.4 ng/mL (Figure 3C), indicating that a high methanol concentration adversely affects the analytical method. When the concentration of methanol was 2.5%, the LOD reached a minimum level of 0.56 ng/mL. Therefore, 2.5% methanol is the best condition for subsequent experiments.

#### 2.3.4. Establishment of A Standard Curve

**Figure 4:**
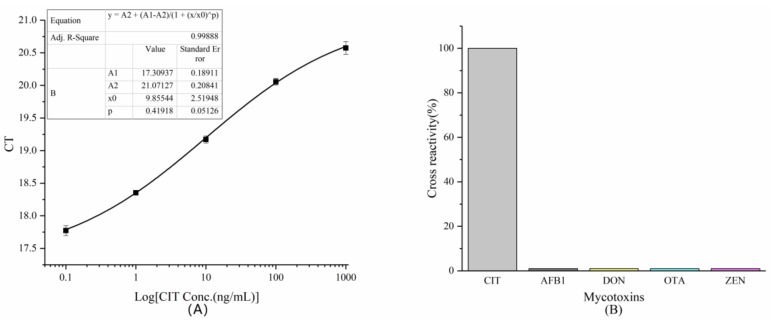
(**A**) The standard curve of quantitative determination of CIT by rtIPCR. (**B**) Cross-reactivity of rtIPCR with four mycotoxins.

Using the optimal conditions obtained from the above experiments, such as the amount of coated anti-CIT McAb, phage input, and methanol concentration, an indirect competition-based rtIPCR method was established to detect the CIT. The number of phages was inversely proportional to the C_T_ level. The IC_50_ of phage-based rtIPCR was 9.86 ± 2.52 ng/mL, with the linear range from 0.1 to 1000 ng/mL, and the LOD was 0.08 ng/mL (Figure 4A). The correlation coefficient of the standard curves was 0.998, which demonstrates the accuracy of the quantification method.

#### 2.3.5. Cross-Reactivity

To verify the specificity of the rtIPCR method established by phage X27 for quantitative detection of CIT, four common mycotoxins—namely, aflatoxin B_1_ (AFB_1_), deoxynivalenol (DON), ochratoxin A (OTA), and zearalenone (ZEN)—were used to perform rtIPCR in place of CIT standards. The cross-reaction rates of this method for these four toxins were calculated and the results are shown in Figure 4B. The rtIPCR method established in the article has no cross-reaction to AFB1, DON, OTA, and ZEN toxins (the cross-reactivity rates are all lower than 0.1%), indicating that the method has good specificity for quantitative detection of CIT.

### 2.4. Assay Validation

To evaluate the reliability of the rtIPCR method established in this paper, a certain amount of CIT standards was added to negative rice and wheat samples. After methanol extraction, the CIT concentration was detected by rtIPCR. The CIT content, spike recovery, and coefficient of variation were calculated for each group. Three replicates of spiked samples were analyzed in one day to evaluate the repeatability of the method, and the results are shown in Table 1. Average recoveries ranged from 90.0 to 104.6% and the coefficient of variation ranged from 6.7 to 14.8% in rice flour; by comparison, average recoveries ranged from 75.8 to 110.0% and the coefficient of variation ranged from 12.3 to 14.3% in flour. The data show that the rtIPCR method established was suitable for the detection of CIT in cereal samples with good recovery, and the lower coefficient of variation (less than 15%).

### 2.5. Sample Analysis

**Figure 5:**
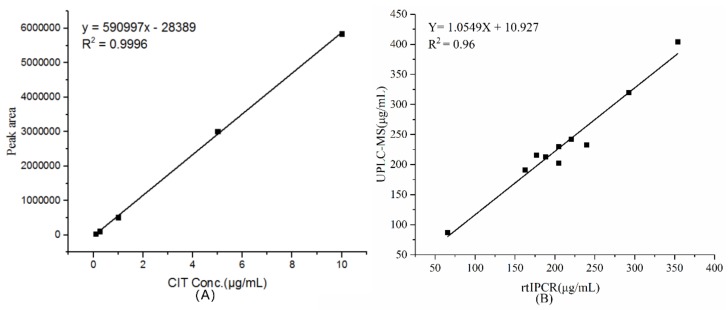
(**A**) The standard curve of quantitative determination of CIT by the UPLC–MS method. (**B**) Comparison of results of rtIPCR method and UPLC–MS method.

Spiked or naturally occurring samples were investigated, and UPLC–MS was performed to verify the results of the rtIPCR. The CIT standard was configured into 0.1, 0.25, 1, 5, and 10 μg/mL solutions for UPLC–MS detection and a standard curve was prepared. The results are shown in Figure 5A. The content of CIT was proportional to the peak area. The linear equation is Y = 590997X − 28389 (Y: Peak area, X: CIT concentration), linear regression analysis revealed a good correlation (*R*^2^ = 0.9996). Then, after all membrane samples of the *Monascus* fermentation broth were diluted 50-fold with methanol, UPLC–MS detection was performed under the same conditions, and the measured CIT contents of each group were calculated according to the formula of the standard curve. After the extracted sample was diluted 1000-fold, the CIT content was determined by rtIPCR and calculated. Concordant results were obtained between rtIPCR and UPLC–MS and linear regression analysis revealed a good correlation between two methods (*R*^2^ = 0.96) (Figure 5B). The result of the paired *t*-test (*p* = 0.536) shows no significant difference between these two methods. Therefore, anti-idiotypic nanobody obtained in this study can functionally mimic antigen for CIT and act as a surrogate antigen for immunoassay.

## 3. Discussion

An immunogen with a molecular weight of less than 1000 is called a hapten. For example, CIT has a molecular weight of 250.25. Such small molecule compounds are not immunogenic, but are reactive. Immunological detection of CIT includes direct competition ELISA and indirect competition ELISA [32,33], in which the former uses CIT coupled with an enzyme as an enzyme-labeled antigen, and the latter uses CIT coupled to a carrier protein as a coating antigen. These two types of antigens act as competing antigens, and their synthesis involves the use of organic reagents and toxin standards, which pollute the environment and endanger the health of operators. Polypeptides and nanobodies can be used as mimics of toxins, directly as coating antigens, and can also be used as enzyme-labeled antigens by fusion expression with AP enzymes [34,35]. Phage displaying the above polypeptide or nanobody can be directly used as a competitive antigen for detecting small molecule compounds. For the first time, Guo et al. applied phage to rtIPCR that greatly simplified the procedure and increases the sensitivity of traditional ELISA methods by up to 10,000 times [36].

The quality of the toxin-carrier protein conjugate greatly affects the sensitivity of detection. The synthesis quality of this type of artificial antigen has a batch error, which has a great influence on the stability of the result. It is necessary to establish a standard curve at the same time for each ELISA. Polypeptides and nanobodies can be obtained by using an *Escherichia coli* expression system and are of stable quality. We must use standards when synthesizing toxin-carrier protein conjugates, but usually, toxin standards are expensive. The polypeptides and nanobodies obtained by expression in *E. coli* are not only inexpensive, but are also available in large quantities. As a mimetic antigen for CIT, a nanobody is not only cheap, but stable with no pollution. Finally, we established an environmentally-friendly immunological analysis method.

The rtIPCR method for the establishment of anti-idiotypic antibody based on phages display has a linear range of 0.1–1000 ng/mL and LOD of 0.08 ng/mL, which is 15 times improved than that of phage ELISA (1.2 ng/mL) in our previous work [30]. Compared with the phage ELISA, the linear range of the method is broadened by about 10 times, although the IC_50_ value is not significantly changed. Lei et al. described the rtIPCR method as follows: The McAb is coated into the enzyme plate for routine phage ELISA experiments, and the eluted phage is subjected to rtIPCR assay instead of traditional ELISA method to quantitatively detect aflatoxin in the grain and feed [37]. Compared to TLC, the recovery rate of the actual sample is higher, and the minimum detection limit is broadened by about 5 times [17]. In regard to biosensors, the minimum detection limit is increased by about 10,000 times [25]. Moreover, our method is more environmentally-friendly and less expensive than HPLC [19]. The detection of CIT in *Monascus*-rice or *Monascus* products requires a maximum dilution of about 180-fold, and the LOD level is 20 ng/mL [33]. Compared to the above gold nanoparticle immunostrip assay, our method is the more sensitive with the larger linear range. The rtIPCR method based on phage displayed anti-idiotypic antibodies has high sensitivity and a broad linear detection range.

## 4. Conclusions

Based on the CIT mimetic antigen phage X27, we established an rtIPCR method for quantitative analysis of CIT from the viewpoint of improving the sensitivity of the immunoassay method. The IC50 value of this method for quantitative detection of CIT was 9.86 ± 2.52 ng/mL, and the linear range was 0.1–1000 ng/mL. RtIPCR showed high sensitivity and broad linear range compared with phage ELISA. The method was successfully validated through UPLC/MS for CIT detection. Overall, rtIPCR could be a reliable and environmentally-friendly method for CIT detection in *Monascus* products.

## 5. Materials and Methods

### 5.1. Chemicals and Reagents

CIT, AFB1, DON, OTA, ZEN, and other toxin standards were purchased from Sigma. Yeast extract was purchased from OXOID. Agarose was purchased from Biowest. SYBR premixes Ex Taq and rTaq were purchased from TaKaRa company. The anti-CIT McAb, CIT mimic-antigen X27, and *E. coli* TG1 cells were prepared by our laboratory. M13K07 helper bacteriophage was a gift from Dr. Greg Winter.

### 5.2. Amplification and Purification of Phage Displaying Anti-Idiotypic Nanobodies

TG1 cells containing the phagemid pHEN-X27 were streaked onto LB-A plates and incubated at 37 °C overnight. The next day, individual colonies were picked out and inoculated into 5 mL of 2× YT-A liquid (16 g/L Tryptone, 10 g/L Yeast extract, 5 g/L NaCl, 100 μg/mL ampicillin, pH 7.0) at 37 °C, 220 rpm for 6 h. The culture was transferred to 25 mL 2× YT-A medium at 37 °C, 220 rpm. Absorbance at 450 nm was determined on a microplate reader. According to the ratio 1:20 of bacteria to helper phage, M13K07 helper phages were added to the bacterial solution at 37 °C for 15 min and transferred to a 37 °C shaker for 30–45 min. The bacteria were centrifuged and the supernatant was discarded. The cell was resuspended in 25 mL of 2× YT-AK liquid (2× YT liquid medium containing 100 μg/mL ampicillin and 50 μg/mL kanamycin), cultured at 30 °C, 220 rpm for 6 h. The bacteria culture was centrifuged and the supernatant was incubated with 1/5 vol of PEG/NaCl (20% PEG 8000, 2.5 M NaCl) for at least 4 h on ice-water. Phages were precipitated by centrifugation and supernatants were discarded. Phages were resuspended in PBS buffer, followed by mixing with an equal vol of glycerol. Phages were stored at −20 °C. 

### 5.3. Phage Titer Determination

First, 10 μL of phage was diluted with 2× YT liquid medium, and 10 μL of each dilution was added to 90 μL of TG1 culture in the logarithmic growth phase and placed at 37 °C for 15 min. Then, the cells were spread on 2× YT-A plates and cultivated at 37 °C overnight. The next day, the colonies were counted and the phage titer was calculated according to the dilution.

### 5.4. PCR Validation of Primer

The plasmid pHEN-X27 and phage X27 were used as templates, and primers 219 (ACGGTGTTTCTGCAAATGAGC) and 220 (GCCCCAGTAGTCATATCGAC) were designed for amplifying the CDR3 region of the mimic-antigen X27. Each PCR reaction system consisted of 2 μL 10× Buffer, 1 μL 10 μM of 219, 1 μL 10 μM 220, 1 μL of plasmid pHEN-X27 or phage X27, 1 U Taq, and 12.5 μL sterilized water. The total volume was 20 µL. The amplification curve was determined using the following process: Initial denaturation at 95 °C for 10 min, followed by 30 cycles of 95 °C for 1 min, 57 °C for 30 s, and 72 °C for 30 s. The PCR products were electrophoresed in 2% agarose gel and observed under the UV imager.

### 5.5. Efficiency Assessment of Real-Time PCR

The gradient annealing temperature was set from 55 to 65 °C. Each column set different the number of gradient phages (10^10^, 10^9^, 10^8^, 10^7^, 10^6^, 10^5^, 10^4^, 10^3^, 10^2^, 10, 0). The phage X27 was used as a DNA template for the PCR. Real-time PCR was performed directly in a 96-well PCR plate using the fast real-time PCR system. Each PCR reaction (20 μL) consisted of 2× SYBR Premix Ex Taq (TaKaRa, Dalian, China), 10 μM of 219 and 220, phage displayed anti-idiotypic antibody nanobodies, and sterilized water. The thermal cycle conditions included heating to 95 °C for 10 min, followed by 40 cycles at 95 °C for 10 s, 55–65 °C for 30 s, and 72 °C for 30 s. Melt curve and CT value were analyzed after data collection.

### 5.6. Phage-Mediated rtIPCR

The optimal conditions for the rtIPCR method were investigated by evaluating the relationship between C_T_ values obtained by rtIPCR and concentration of CIT standard. Each experiment was performed under various conditions (coated anti-CIT McAb concentration, phage dose, and methanol concentration). All experiments were conducted in triplicate. Anti-CIT McAb (1.5 μg/mL, 100 mL/well) was coated on 96-well microtiter plate at 4 °C overnight, and the wells were washed four times with PBST (PBS, containing 0.05% Tween-20). After blocking with 4% skimmed milk-PBS for 1 h at 37 °C and washing four times with PBST, 50 μL of 10% methanol-PBS to prepare gradient dilutions (0.1, 1, 10, 100, 1000 ng/mL) of CIT standard and 50 μL phage were added to the wells and mixed, and the mixture was incubated at 37 °C for 1 h. After washing four times with PBST, the bound phages were eluted by Gly–HCl (7.5 g/L glycine, pH 2.2) in a horizontal shaker at room temperature for 10 min and neutralized with Tris–HCl (121 g/L Tris, pH 9.0). The eluted phages were used as a DNA template for the real-time PCR. The real-time PCR was performed in a 96-well PCR plate in the CFX Connect real-time PCR system (Bio-Rad, Hercules, CA, USA). Each PCR reaction (20 μL) consisted of 2× SYBR Premix Ex Taq, 10 μM of primers 219 and 220, 6 μL of eluted phage, and sterilized water. The thermal cycle was performed at 95 °C for 10 min, followed by 40 cycles at 95 °C for 10 s, 62 °C for 30 s, and 72 °C for 30 s. Melt curve and C_T_ value were analyzed after data collection.

### 5.7. Spike and Recovery Analysis

To evaluate the accuracy of the rtIPCR method, spiking and recovery experiments were implemented. Samples of rice flour and flour from local markets (Nanchang, China) were finely ground, and 2g of the weighed ground samples, shown to be CIT-free by HPLC, were fortified with 0.1, 1, 10, 100, and 1000 μg/kg CIT standard, respectively, and mixed thoroughly for 15 min. For the extraction, 10 mL of 5% methanol-PBS solution was added and the tubes were vigorously shaken for 30 min. Then, the samples were centrifuged at 8000 rpm for 20 min and the supernatant was filtered through a 0.22 μm cellulose acetate membrane. The concentration of CIT in the sample extracts were analyzed by rtIPCR assay.

### 5.8. Sample Analysis

*Monascus* spores were inoculated in YES medium 80 g/L sucrose, 20 g/L yeast extract), and cultured in a constant temperature incubator at 28 °C for 10 days. Next, 500 μL of *Monascus* fermentation broth was placed in a centrifuge tube and centrifuged at 5000 rpm for 10 min to discard the pellet. An equal volume of methanol was added to the supernatant. Then, the mixture was ultrasonically-assisted extracted for 30 min and continued to extract at room temperature for 6–8 h, then centrifuged at 10,000 rpm for 20 min. The supernatant was filtered through a 0.45 μm organic membrane. LC–MS analysis of CIT was carried out on a Waters ZQ 4000/2685 HPLC–MS system. The separation was performed on a Shim-pack GIST C18 column (2.1 × 75 mm, 2 μm), the mobile phase A consisted of 0.1% (*v*/*v*) formic acid in the water, and the mobile phase B comprised acetonitrile. The flow rate of mobile phases was 0.2 mL/min, and the injection volume was 5 µL. The temperature of the thermostatic column compartment was maintained at 30 °C. The mass spectrometer was operated in a negative electrospray ionization (ESI) mode. The ionization conditions were as follows: Capillary voltage, 3 kV; gas temperature, 350 °C; nebulizer gas pressure, 65 psi; nitrogen flow rate, 11 mL/min. At the same time, the above samples were detected by rtIPCR. The data obtained from the two groups were analyzed by a paired *t*-test. The difference between individual means was determined using a least significant difference (LSD) test. The statistical significance was set at *p* = 0.05. Data analyses were performed using SPSS software (IBM SPSS Statistics Subscription, IBM, New York, NY, USA).

## Figures and Tables

**Table 1:** Results of recovery experiment of rtIPCR.

Sample	CIT Add Amount (μg/kg)	rtIPCR (*n* = 3)		
		Average Content ± Deviation (μg/kg)	Recovery Rate (%)	Coefficient of Variation (%)
Rice flour	0.1	0.09 ± 0.01	90.0	11.2
	1	1.05 ± 0.16	104.6	14.8
	10	10.4 ± 1.0	103.9	9.8
	100	97.3 ± 6.5	97.3	6.7
	1000	916.4 ± 116.6	91.6	12.7
Flour	0.1	0.09 ± 0.01	85.6	14.3
	1	1.10 ± 0.14	110.0	13.2
	10	9.5 ± 1.2	94.7	12.3
	100	102.9 ± 12.9	103.0	12.5
	1000	757.9 ± 93.1	75.8	12.3
